# Live Attenuated B. pertussis as a Single-Dose Nasal Vaccine against Whooping Cough

**DOI:** 10.1371/journal.ppat.0020065

**Published:** 2006-07-07

**Authors:** Nathalie Mielcarek, Anne-Sophie Debrie, Dominique Raze, Julie Bertout, Carine Rouanet, Amena Ben Younes, Colette Creusy, Jacquelyn Engle, William E Goldman, Camille Locht

**Affiliations:** 1 INSERM U629, Lille, France; 2 Institut Pasteur de Lille, Lille, France; 3 IFR142, Lille, France; 4 Service d'Anatomie et de Cytologie Pathologique, Groupe Hospitalier de l'Institut Catholique de Lille, Faculté Libre de Médecine, Lille, France; 5 Washington University, St. Louis, Missouri, United States of America; Scripps Research Institute, United States of America

## Abstract

Pertussis is still among the principal causes of death worldwide, and its incidence is increasing even in countries with high vaccine coverage. Although all age groups are susceptible, it is most severe in infants too young to be protected by currently available vaccines. To induce strong protective immunity in neonates, we have developed BPZE1, a live attenuated Bordetella pertussis strain to be given as a single-dose nasal vaccine in early life. BPZE1 was developed by the genetic inactivation or removal of three major toxins. In mice, BPZE1 was highly attenuated, yet able to colonize the respiratory tract and to induce strong protective immunity after a single nasal administration. Protection against B. pertussis was comparable to that induced by two injections of acellular vaccine (aPV) in adult mice, but was significantly better than two administrations of aPV in infant mice. Moreover, BPZE1 protected against Bordetella parapertussis infection, whereas aPV did not. BPZE1 is thus an attractive vaccine candidate to protect against whooping cough by nasal, needle-free administration early in life, possibly at birth.

## Synopsis

Although vaccination has strongly reduced the incidence of whooping cough in many countries, this disease still causes approximately 300,000 deaths per year, mainly in young children that are not fully vaccinated. Efficient protection against pertussis requires at least three vaccine doses and is not achieved before the age of 6 mo. A new strategy to induce strong protective immunity in neonates is to mimic as closely as possible natural infection without inducing the disease, by the use of a live attenuated B. pertussis strain to be given as a single-dose nasal vaccine. The authors examined in the mouse model the efficacy of a genetically attenuated strain, BPZE1. This strain colonizes the mouse respiratory tract, but appears to be highly attenuated as evidenced by histopathological studies. In addition, a single nasal administration of this strain protects against challenge with virulent B. pertussis better than two administrations of acellular vaccine in infant mice. Moreover, BPZE1 provides protection against infection with Bordetella parapertussis responsible for a milder pertussis-like syndrome, which was not seen after vaccination with acellular vaccine. These results show that BPZE1 could be an efficient, single-dose nasal vaccine to protect early in life against whooping cough.

## Introduction

Whooping cough, or pertussis, is a severe childhood disease responsible for high mortality rates before the introduction of effective vaccines in the second half of the 20th century. The success of these vaccines has led to the opinion that the disease is essentially under control, although worldwide 200,000 to 400,000 pertussis-linked deaths are still recorded annually, and the disease still ranks sixth among the causes of mortality due to infectious agents [1]. Although mostly prevalent in developing countries, the disease is also re-emerging in the developed world [2,3], including the United States, where the incidence has increased nearly 10-fold over the last 20 y [4]. Unexpectedly, the epidemiology of pertussis has changed in countries with high vaccine coverage, where cases of adolescent and adult pertussis are increasingly frequent [5]. This is probably due to progressive waning of vaccine-mediated immunity during adolescence. Often atypical and therefore difficult to diagnose, pertussis is generally not life-threatening in adults and in many cases remains unnoticed. However, infected adults constitute an important reservoir for transmission of the disease to very young children, too young to be fully vaccinated, and therefore at risk to develop severe disease associated with high mortality rates.

Pertussis vaccination usually begins at 2 mo of age, and optimal protection requires at least three immunizations. Generally, the three doses are given at 1- to 2-mo intervals, implying that optimal protection is only achieved at the age of 6 mo. To reduce the incidence of pertussis in the very young and most vulnerable age groups, early immunization, possibly at birth, would thus be highly desirable. However, numerous studies in humans and in animal models have suggested that the neonatal immune system is too immature to effectively induce vaccine-mediated protective immunity [6,7]. The IFN-γ production especially, which is indicative of a Th1 response that is essential to the development of protective immunity to pertussis [8], appears to be significantly reduced in human newborns, compared to older children or adults [9]. This is also reflected by the fact that significant amounts of antigen-specific IFN-γ are only produced after several months (≥6 mo) in children vaccinated with pertussis vaccines, especially with acellular vaccines (aPV) [10].

Natural infection with Bordetella pertussis has long been considered to induce strong and long-lasting immunity that wanes later than vaccine-induced immunity [5,11]. Furthermore, infection with B. pertussis induces measurable antigen-specific Th1-type immune responses even in very young children (as young as 1 mo of age) [12]. These observations suggest that live vaccines applicable by the nasal route, in order to mimic as closely as possible natural infection, may be attractive alternatives over the currently available vaccines.

In this report, we describe the development of such a live vaccine candidate through genetic attenuation of B. pertussis to diminish pathogenicity while maintaining the ability to colonize and induce protective immunity. This vaccine candidate was found to induce protection in infant mice after a single intranasal administration that is superior to the protection provided by the current aPV. In addition, it provided protection against infection with *Bordetella parapertussis,* which was not seen after vaccination with aPV.

## Results

### Construction of B. pertussis BPZE1

Recent advances in the understanding of B. pertussis virulence at the molecular level [13] have allowed us to rationally design a strategy for attenuation by removing or altering genes that are involved in the pathogenesis of whooping cough. Three virulence factors were genetically targeted: tracheal cytotoxin (TCT), pertussis toxin (PTX), and dermonecrotic toxin (DNT).

**Figure 1:**
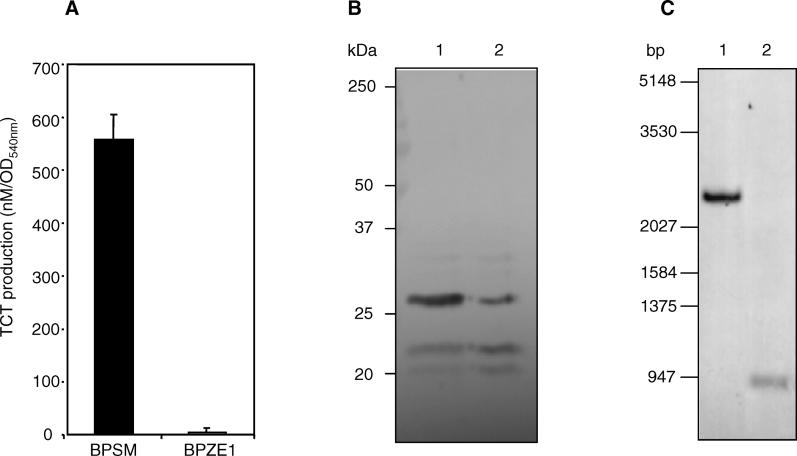
Characterization of B. pertussis BPZE1 (A) TCT present in culture supernatants of BPSM and BPZE1 expressed as means of nM/OD_540nm_ (± standard error) of three separate cultures for each strain. (B) Immunoblot analysis of PTX production in the culture supernatants of BPSM (lane 1) and BPZE1 (lane 2). The sizes of the molecular weight (M*r*) markers are expressed in kDa and given in the left margin. (C) Southern blot analysis of the *dnt* locus in BPSM (lane 1) and BPZE1 (lane 2). The lengths of the size markers are indicated in base pairs (bp) are shown in the left margin.

TCT is responsible for the destruction of ciliated cells in the trachea of infected hosts [14,15] and may thus be involved in the cough syndrome. TCT is a breakdown product of peptidoglycan in the cell wall of Gram-negative bacteria, which generally internalize it into the cytosol by the AmpG transporter protein to be re-utilized during cell wall biosynthesis. B. pertussis AmpG is inefficient in the internalization of peptidoglycan breakdown products. We therefore replaced the *B. pertussis ampG* gene by *E. coli ampG*. The resulting strain expressed less than 1% residual TCT activity (Figure 1A).

PTX is a major virulence factor responsible for the systemic effects of B. pertussis infections and is composed of an enzymatically active moiety, called S1, and a moiety responsible for binding to target cell receptors (for review, see [16]). However, it is also one of the major protective antigens, which has prompted us to replace the natural *ptx* genes by a mutated version coding for an enzymatically inactive toxin. This was achieved by replacing Arg-9 by Lys, and Glu-129 by Gly in S1, two key residues involved in substrate binding and catalysis, respectively. Allelic exchange was used to first delete the *ptx* operon, and then to insert the mutated version. The presence of the relevant toxin analogs in the B. pertussis culture supernatants was detected by immunoblot analysis, although the mutant strain consistently produced slightly lower levels of anti-PTX antibody reactive material than the parental strain (Figure 1B).

Finally, allelic exchange was used to remove the *dnt* gene (Figure 1C). Although the role of DNT in the virulence of B. pertussis is not certain, it has been identified as an important toxin in the closely related species Bordetella bronchiseptica and displays lethal activity upon injection of minute quantities (for review, see [16]).

### In Vitro Characterization of B. pertussis BPZE1

**Figure 2:**
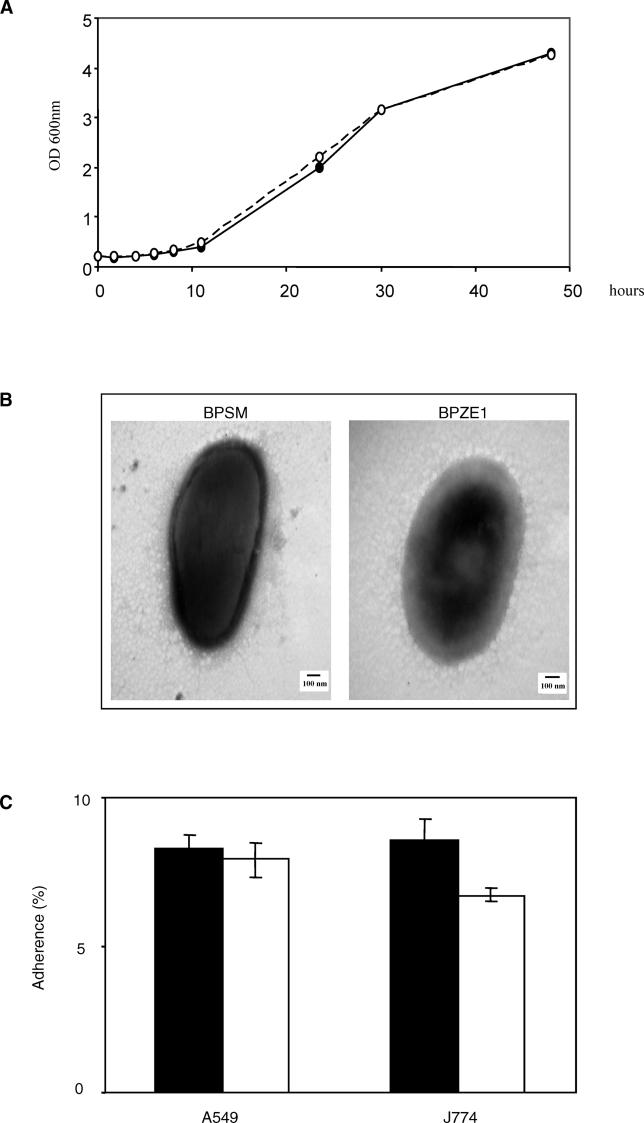
Phenotypic Characterization of B. pertussis BPZE1 (A) Growth rates of BPSM (solid line) and BPZE1 (dotted line) in liquid culture. (B) Electron micrographs representative of BPSM (left) and BPZE1 (right) grown in liquid medium for 24 h. (C) In vitro adherence of BPSM (filled columns) and BPZE1 (open columns) to human pulmonary epithelial A549 cells (left) and murine macrophage-like J774 cells (right). The results are expressed as means (± standard error) of percentages of binding bacteria relative to the bacteria present in the inoculum from three different experiments.

Since some of the genetic alterations in BPZE1 may potentially affect the bacterial cell wall synthesis, we compared the size and shape, as well as the in vitro growth rate, of BPZE1 with those of the parental strain BPSM (see Materials and Methods for reference). The growth rate of BPZE1 did not differ from that of BPSM (Figure 2A), and no difference in bacterial shape or size was detected between BPZE1 and BPSM, as evidenced by electron microscopy analysis (Figure 2B). However, the cell wall of BPZE1 appeared to be consistently somewhat thinner than that of BPSM.

To determine whether the absence or alterations of any of the targeted toxins in BPZE1 affect adherence properties of *B. pertussis,* we compared the attachment rates of BPZE1 with those of BPSM, using the human pulmonary epithelial cell line A549 and the murine macrophage cell line J774, two cellular models often used to study the adherence of B. pertussis. No statistically significant difference in the adherence capacities to either cell line was observed between the two strains (Figure 2C).

### Attenuation of B. pertussis BPZE1

**Figure 3:**
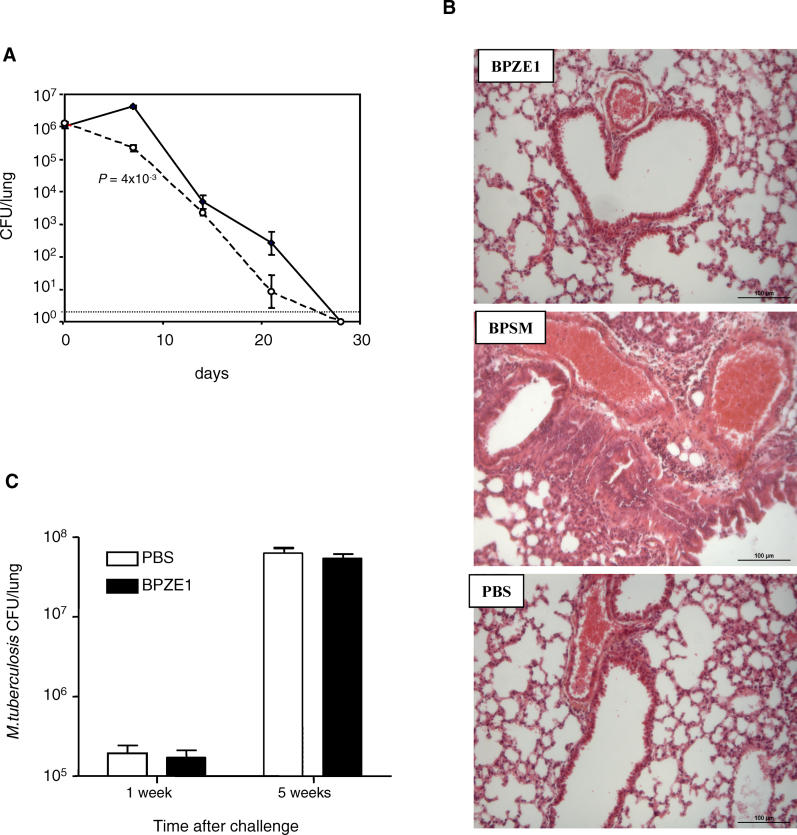
In Vivo Characterization of B. pertussis BPZE1 (A) Lung colonization by BPSM (solid lines) and BPZE1 (dotted lines) of adult mice infected intranasally with 10^6^ CFU of BPZE1 or BPSM. The results are expressed as mean (± standard error) CFUs from three to four mice per group, and are representative of two separate experiments. The dashed line represents the limit of bacterial counts. (B) Histological analysis of lungs from BPZE1 (upper panel) or BPSM-infected (middle panel) adult mice compared to controls given PBS (lower panel). One week after infection, the lungs were aseptically removed and fixed in formaldehyde. Sections were stained with hematoxylin and eosin, and examined by light microscopy. (C) Susceptibility of BPZE1-infected mice to infection by M. tuberculosis. Balb/C mice were infected intranasally with 10^6^ CFU of BPZE1 (filled columns) or received PBS (open columns) and were intranasally infected one week later with 5 × 10^4^ M. tuberculosis H37Rv. One week (left columns) and 5 wk (right columns) after M. tuberculosis infection, the M. tuberculosis CFUs present in the lungs were counted. The results are expressed as mean (± standard error) CFUs from four mice per group.

To determine whether the mutations introduced into B. pertussis BPZE1 have resulted in attenuation, yet allow the organism to colonize the respiratory tract, 8-wk-old Balb/C mice were intranasally infected with BPZE1 or BPSM, and colonization was followed over time. BPZE1 was able to colonize and persist in the lungs of mice as long as BPSM (Figure 3A**)**. However, the peak of multiplication seen 7 d after infection with BPSM was consistently lacking in mice infected with BPZE1. Studies done with strains mutated in individual toxin genes indicated that this is due to the mutations in the *ptx* locus (unpublished data). When the lungs were examined for histopathological changes and inflammatory infiltration, infection with BPSM was found to induce strong peri-bronchiovascular infiltrates and inflammatory cell recruitment 7 d after infection, associated with a strong hypertrophy of the bronchiolar epithelial cells (Figure 3B). In contrast, no such changes were seen in BPZE1-infected animals, and the histology of the BPZE1-infected mice was similar to that of the control mice that had received sterile phosphate-buffered saline (PBS) instead of the bacteria. Similar results were obtained when infant mice (3 wk old) were used instead of adult mice (see Figure S1) The BPSM infection–induced inflammation lasted for at least 2 mo (unpublished data). These results indicate that the mutations introduced into BPZE1 have resulted in drastic attenuation, but allow the bacteria to colonize and persist for up to 1 mo.

To test whether intranasal vaccination with BPZE1 may predispose mice to other respiratory infections, BPZE1-vaccinated mice were infected with *Mycobacterium tuberculosis,* the most prevalent bacterial pathogen of the respiratory tract, and the mycobacterial colony counts were determined 1 and 5 wk after infection. No significant difference was observed between mice immunized with BPZE1 prior to M. tuberculosis infection and those that had received PBS instead of BPZE1 (Figure 3C).

### Protection against B. pertussis Challenge after Intranasal Vaccination of Adult Mice with BPZE1

**Figure 4:**
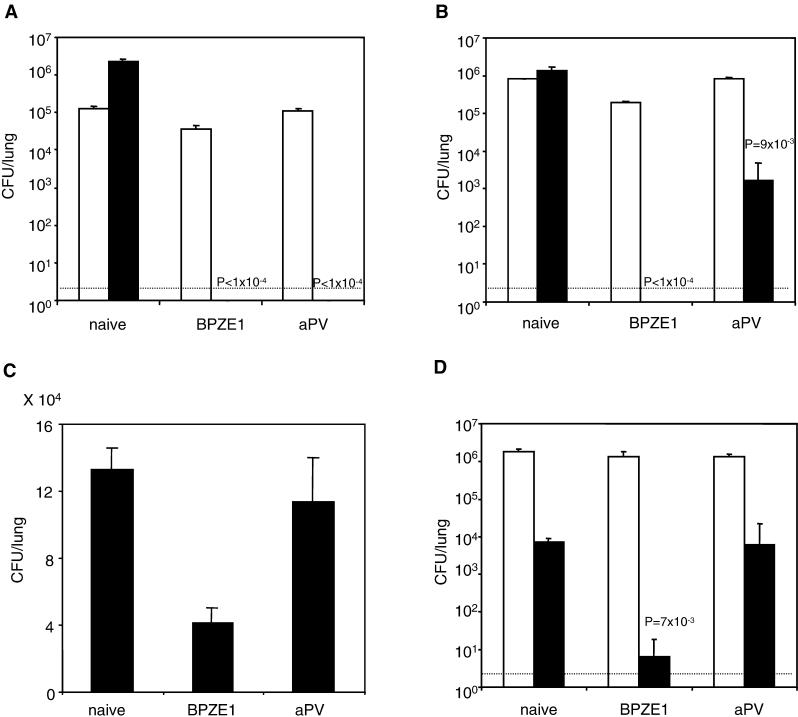
Protection against *Bordetella* Infection Protection against B. pertussis in adult (A) and infant mice (B) and (C), or against B. parapertussis in infant mice (D). Mice immunized with BPZE1, aPV, or PBS (naive) were challenged with BPSM (A), (B), and (C), or B. parapertussis (D), and lung CFU counts were determined 3 h (open columns) or 7 d (filled columns) later. Results are expressed as mean (± standard error) CFUs from three to four mice per group and are representative of two separate experiments. (C) CFU counts 3 h after BPSM challenge in adult mice vaccinated with BPZE1 or aPV, compared to controls. The dashed lines in panels (A), (B), and (D) represent the limit of bacterial counts.

To evaluate the protection offered by BPZE1, we compared the effect of a single intranasal administration of this strain to 8-wk-old Balb/C mice on the subsequent colonization by the wild-type challenge strain BPSM with that of two intraperitoneal (ip) immunizations with 1/5 of a human dose of aPV. This aPV immunization protocol has been described as the best to correlate with pertussis vaccine efficacy in human clinical trials [17,18]. As shown by the total clearance of bacterial colony counts in the lungs 7 d after challenge infection, a single intranasal administration of BPZE1 and two ip immunizations with aPV provided similar levels of protection (Figure 4A). High bacterial loads were found in the control mice that had received two injections of PBS instead of the vaccine.

### Protection against B. pertussis Challenge after Intranasal Vaccination of Infant Mice with BPZE1

Since the principal targets of novel pertussis vaccines are young infants, which are not protected with the currently available vaccines, we have developed an infant (3-wk-old) mouse model [19] and used it here to compare the efficiency of vaccination with BPZE1 with that of vaccination with aPV. A single nasal administration of BPZE1 fully protected infant mice against challenge infection (Figure 4B), as complete bacterial clearance was observed in the lungs 1 wk after challenge. In contrast, substantial numbers of bacteria remained in the aPV-vaccinated animals 1 wk after challenge infection. The difference in bacterial load between the BPZE1-vaccinated and the aPV-vaccinated mice was statistically significant, indicating that in the infant mouse model, a single intranasal administration with BPZE1 provides better protection than two systemic administrations of aPV.

In addition, we consistently observed a strong reduction in the bacterial load of the challenge strain 3 h after administration when the mice had been immunized with BPZE1 compared to the aPV-immunized animals (Figure 4C), indicating that vaccination with BPZE1 reduces the susceptibility to infection by the challenge strain. This effect was seen in both 8-wk-old and in infant mice. In contrast, aPV had no effect on the bacterial counts 3 h after infection, when compared to the control mice.

### Protection against B. parapertussis Challenge after Intranasal Vaccination with BPZE1

There is increasing concern about B. parapertussis infection in children, especially in immunized populations [20,21]. B. parapertussis causes a milder pertussis-like syndrome, the frequency of which is probably largely underestimated. Furthermore, the incidence of B. parapertussis infections has been increasing over the last decades, possibly due to the fact that pertussis vaccines are known to have low or no protective efficacy against B. parapertussis [22,23]. In contrast, infection by B. pertussis has recently been reported to protect against B. parapertussis infection in the mouse model [24]. We therefore assessed BPZE1 for protection against B. parapertussis using the infant mouse model. Whereas two administrations of aPV did not provide any protection against *B. parapertussis,* a single intranasal administration of BPZE1 provided strong protection, as measured by the low numbers of B. parapertussis counts in the lungs of the vaccinated mice 1 wk after challenge (Figure 4D).

### Immune Responses Induced by BPZE1 Vaccination

**Figure 5:**
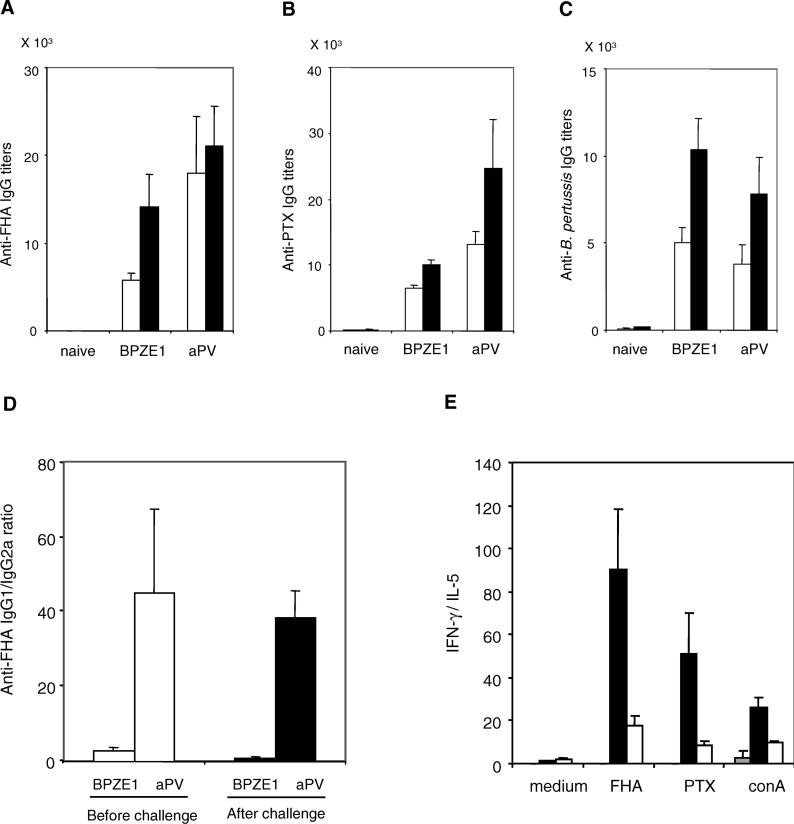
Immune Responses Induced in Infant Mice by BPZE1 or aPV Immunization (A) Anti-FHA, (B) anti-PTX, (C) anti-B. pertussis IgG heavy and light chain (H+L) titers, and (D) anti-FHA IgG1/IgG2a ratios before (open columns) or 1 wk after BPSM challenge (filled columns) in BPZE1 or aPV immunized 3-wk-old mice, compared to controls. (E) IFN-γ to IL-5 ratios produced by FHA-, PTX- or ConA-stimulated splenocytes from 8-wk-old mice vaccinated 2 mo before with BPZE1 (filled columns) or aPV (open columns), compared to controls (gray columns). Antibodies and cytokines were measured in individual mice, and the results are expressed as mean values (± standard error) for four mice per group tested in triplicate.

Although the mechanisms of protective immunity against B. pertussis infection are not yet completely understood, clear evidence of a role for both B cells and IFN-γ has been demonstrated in mice [18]. Vaccination of infant mice with either one nasal dose of BPZE1 or two ip administrations of aPV induced high titers of serum immunoglobulin G (IgG) against filamentous hemagglutinin (FHA) (Figure 5A), a major surface antigen of B. pertussis [25], and against PTX (Figure 5B), both present in aPV, as well as against a B. pertussis whole cell lysate (Figure 5C). Following B. pertussis challenge, positive anamnestic responses were measured in BPZE1- and in aPV-vaccinated animals, as indicated by an increase in anti-FHA, anti-PTX, and anti-B. pertussis whole cell lysate IgG titers, compared to primary responses before B. pertussis infection. Similar results were obtained in 8-wk-old mice, as shown for the anti-FHA responses in Figure S2. Examination of the anti-FHA IgG1/IgG2a ratios showed that these ratios were higher after aPV administration, characteristic of a Th2-type response, than after BPZE1 vaccination (Figure 5D). After challenge, the anti-FHA-IgG1/IgG2a ratio remained substantially higher in the aPV-vaccinated mice than in the BPZE1-vaccinated animals.

Analysis of B. pertussis antigen-specific cytokine patterns induced by BPZE1 or aPV vaccination of 8-wk-old mice confirmed that BPZE1 administration favors a stronger Th1-type response than aPV vaccination. This was revealed by the fact that the ratios of IFN-γ over IL-5 produced by splenocytes stimulated with FHA or PTX, or with the polyclonal activator ConA were significantly higher in BPZE1 vaccinated mice than in aPV vaccinated mice (Figure 5E). For technical reasons, these experiments could only be performed in adult mice.

## Discussion

Pertussis is the first infectious disease whose incidence is increasing in countries with high vaccine coverage, highlighting the limits of current vaccine strategies. The fact that natural B. pertussis infection, even very early in life, is able to induce a strong and long-lasting Th1 response [12] prompted us to develop a live attenuated B. pertussis vaccine strain to be given by the nasal route as an alternative over the currently available vaccines.

On the basis of experimental infections of primates, Huang et al. [26] in 1962 had already come to the conclusion that ultimate protection against whooping cough probably best follows a live B. pertussis inoculation. In veterinary medicine, attenuated *Bordetella* strains have been used to vaccinate against bordetellosis in dogs and piglets. A live attenuated Bordetella bronchiseptica strain has been shown to provide strong protection against kennel cough in dogs [27] after nasal administration. This protection was seen as early as 48 h after vaccination. Intranasal vaccination with live attenuated B. bronchiseptica has also been shown to protect against atrophic rhinitis in 2-d-old piglets [28], indicating that in a live attenuated form, *Bordetella* vaccines can be highly active in newborn animals.

Previous attempts to genetically attenuate B. pertussis as a live vaccine candidate have met with rather limited success. Based on a strategy used for the successful attenuation of *Salmonella* vaccine strains [29], Roberts et al. [30] have deleted the *aroA* gene of B. pertussis. The *aroA* mutant was indeed highly attenuated, but it had also lost its capacity to colonize the respiratory tract of the intranasally vaccinated animals, and induced protective immunity only after repeated administrations of high doses. We took advantage of the knowledge on the molecular mechanisms of B. pertussis virulence and developed the highly attenuated strain BPZE1. This strain contains genetic alterations leading to the absence or inactivation of three major toxins, PTX, TCT, and DNT. In contrast to the *aroA* mutant, this strain was able to colonize the mouse respiratory tract and to provide full protection after a single intranasal administration. The protection in adult mice was indistinguishable from that induced by two administrations of 1/5 of a human dose of aPV. An important difference, however, was seen in infant mice, in which a single administration of BPZE1 fully protected, whereas aPV only offered partial protection. In the context of the difficulties to induce protection in infants early in life with the currently available vaccines, these results provide hope for the development of novel vaccination strategies that may be used in the very young children, possibly at birth. In addition, BPZE1 protected against *B. parapertussis,* whereas aPV did not. Therefore the use of BPZE1 should also have an impact on the incidence of whooping cough caused by B. parapertussis in infants, provided that potential programmatic obstacles to replace the current vaccines with a live attenuated strain can be overcome.

Although the recent replacement of first generation, whole-cell vaccines by new aPV in many countries has significantly reduced the systemic adverse reactions observed with whole-cell vaccines, it has not abolished the need for repeated vaccination to achieve protection. This makes it unlikely to obtain protection in very young children (<6 mo) that present the highest risk to develop severe disease. In addition, the wide-spread use of aPV has revealed new, unforeseen problems. Repeated administration of aPV may cause extensive swelling at the site of injection [31]. In approximately 5% of the cases, this swelling involves almost the entire limb and lasts for more than a week. Although the mechanism of this swelling has not been characterized yet, it has been proposed to be due to an Arthus hypersensitivity reaction caused by high antibody levels induced by the primary immunization [32]. However, it could also be related to the Th2 skewing of the immune response, as, compared to whole-cell vaccines, aPV administration induces more Th2-type cytokines in vaccinated children [10] and causes a delay in the Th1 development (F. Mascart, M. Hainaut, A. Peltier, V. Verscheure, J. Levy, and C. Locht, unpublished data). Delayed maturation of Th1 function has been associated with a risk for atopy in genetically pre-disposed individuals [33]. The two mechanisms are not mutually exclusive. Compared to aPV, the immune response to BPZE1 administration is less biased towards the Th2 arm, and since BPZE1 is administered mucosally, no swelling reaction can occur.

The use of live attenuated bacteria as vaccines raises the issue of their biosafety. As such, they fall under the directives and guidelines for genetically modified organisms susceptible to be released into the environment. These guidelines and directives describe several objectives that have to be met, including hazard identification and environmental risk assessment [34]. Potential pathogenicity needs to be carefully considered, especially in immunocompromised individuals, such as those infected with HIV. The natural biology of B. pertussis is particularly interesting in that regard. Although pertussis in HIV-infected subjects has been described occasionally, it is rather rare in AIDS patients [35]. In its genetically attenuated form, B. pertussis would therefore not be expected to cause major problems in HIV-infected children, especially if severe AIDS is an exclusion criterion, as it is for many vaccines. B. pertussis colonization is strictly limited to the respiratory epithelium, without extrapulmonary dissemination of the bacteria, which naturally excludes systemic bacteremia of the BPZE1 vaccine strain. If nevertheless unforeseeable safety problems occurred, the vaccine strain can be eliminated early following vaccination by the use of macrolide antibiotics, such as erythromycine, to which essentially all B. pertussis isolates are sensitive.

A further concern, as for any live vaccine, is the potential release of the vaccine strain into the environment, and the consequences of such a release. B. pertussis is predominantly a human pathogen, and there is no known animal vector or reservoir. Moreover, unlike *B. bronchiseptica,* survival of wild-type B. pertussis in the environment is extremely limited [36]. Pertussis is usually spread by coughing individuals, and there appears to be limited asymptomatic carriage [37]. Coughing cannot be assessed in the mouse models used in this study. However, due to the nature of the genetic alterations in BPZE1, in particular the strong reduction of TCT and the genetic inactivation of PTX, this strain would not be expected to induce coughing. Active PTX has been shown to be required for cough induction in a coughing rat model, although the mechanism is not known [38]. If the vaccine strain were nevertheless to be transmitted to non-vaccinated individuals, this would at worst result in increased vaccine coverage. The consequences of each of these potential hazards can thus be graded as negligible and can easily and rapidly be controlled by antibiotic treatment if necessary.

Advantages of the use of BPZE1 include the relatively low production costs, making it especially attractive for developing countries, its needle-free, easy, and safe mode of administration, and its potential to induce mucosal immunity in addition to systemic immunity. Although, surprisingly, the role of mucosal immunity against pertussis has not been much addressed, the fact that B. pertussis is a strictly mucosal pathogen, makes it likely that mucosal immune responses may contribute significantly to protection. None of the currently available vaccines induces any significant mucosal response.

The use of live attenuated B. pertussis for mucosal vaccination offers yet another advantage. B. pertussis can be used for the presentation of heterologous antigens to the respiratory mucosa (for review, see [39]) The use of BPZE1 as a heterologous expression platform may thus be helpful for the generation of multivalent vaccines against a variety of respiratory pathogens. However, since intranasal delivery of BPZE1 also induces strong systemic immune responses, as shown here by both the high levels of anti-FHA antibodies and of antigen-specific IFN-γ production, it may also be used for the production of antigens to which systemic immune responses are desired.

## Materials and Methods

### *Bordetella* strains and growth conditions.

The B. pertussis strains used in this study are all derived from B. pertussis BPSM [40], and B. parapertussis is a streptomycin-resistant derivative of strain 12822 (kindly provided by Dr. N. Guiso, Institut Pasteur de Paris, France). All *Bordetella* strains were grown on Bordet-Gengou (BG) agar (Difco Laboratories, Detroit, Michigan, United States) supplemented with 1% glycerol, 20% defibrinated sheep blood, and 100 μg/ml streptomycin. For cell adherence assays, exponentially growing B. pertussis was inoculated at an optical density of 0.15 at 600 nm in 2.5-ml modified Stainer-Scholte medium [41] containing 1 g/l heptakis(2,6-di-*o*-methyl) β-cyclodextrin (Sigma, St. Louis, Missouri, United States) and supplemented with 65 μCi/ml L-[^35^S]methionine plus L-[^35^S]cysteine (NEN, Boston, Massachusetts, United States) and grown for 24 h at 37 °C.The bacteria were then harvested by centrifugation, washed three times in phosphate-buffered saline (PBS), and resuspended in RPMI 1640 (Gibco, Grand Island, New York, United States) at the desired density. M. tuberculosis H37Rv was grown in Sauton medium or on Middlebrook 7H11 agar (Difco Laboratories) at 37 °C.

### Construction of B. pertussis BPZE1.

The different steps followed to construct B. pertussis BPZE1 can be found in Protocol S1. Briefly, we first replaced by allelic exchange the *B. pertussis ampG* gene in the BPSM genome by *Escherichia coli ampG*. Next, allelic exchange was used to delete the *ptx* operon and to then insert a mutated version coding for enzymatically inactive PTX. Finally, the *dnt* gene was deleted from the resulting B. pertussis strain.

### Analysis of TCT production.

For sensitive quantitation of TCT production, culture supernatants of B. pertussis grown to logarithmic phase were collected, subjected to solid phase extraction [42], and derivatized with phenylisothiocyanate (PITC; Pierce Biotechnology, Rockford, Illinois, United States). The resulting phenylthiocarbamyl (PTC) derivatives were separated by reversed-phase high-performance liquid chromatography using a C8 column (Perkin Elmer, Wellesley, California, United States) and detected at 254 nm. The amount of B. pertussis PTC-TCT in each sample was determined by comparing the peak area and elution time with an identically processed TCT standard.

### Cell-adherence assay.

To analyze adherence properties of the B. pertussis strains, their attachment rates to the human pulmonary epithelial cell line A549 (ATCC n CCL-185) and the murine macrophage cell line J774 (ATCC n TIB-67) were measured as previously described [43].

### Transmission electron microscopy.

The single-droplet negative staining procedure was used as described previously [44] with the following modifications: 20 μl of a suspension at approximately 10^9^ bacteria/ml were absorbed for 2 min onto form Formvar carbon-coated nickel grids (400 mesh; Electron Microscopy Sciences EMS, Washington, Pennsylvania, United States). After 30 s air-drying, the grids were stained for 2 min with 20 μl of 2% phosphotungstic acid (pH7) (EMS) and examined after air-drying under a transmission electron microscope (Hitachi 7500; Hitachi, Tokyo, Japan) at 60 kV and high resolution.

### Intranasal infection and vaccination.

The 3-wk-old and 8-wk-old female Balb/C mice were kept under specific pathogen-free conditions, and all experiments were carried out under the guidelines of the Institut Pasteur de Lille animal study board. Mice were intranasally infected with approximately 4 × 10^6^ bacteria in 20-μl PBS, and kinetics of colony-forming units (CFU) in the lungs were measured as previously described [45]. For vaccination with aPV (Tetravac; Aventis-Pasteur, Lyon, France), mice were immunized ip with 20% of the human dose and boosted 1 mo later using the same dose. For challenge infections with *Bordetella,* mice were intranasally infected 2 mo after vaccination with approximately 4 × 10^6^ BPSM or B. parapertussis in 20-μl PBS. For challenge infection with *M. tuberculosis,* mice were intranasally infected with 5 × 10^4^ M. tuberculosis H37Rv in 20-μl PBS 1 wk after vaccination with BPZE1.

### Antibody determination.

Sera were collected, and antibody titers were estimated by enzyme-linked immunosorbent assays (ELISA) as previously described [45].

### Cytokine assays.

Spleen cells from individual mice were tested at different time points after immunization for in vitro cytokine production in response to heat-killed B. pertussis BPSM (10^6^ cells/ml), 5.0 μg/ml PTX (purified from B. pertussis BPGR4 [46] as previously described [47], and heat-inactivated at 80 °C for 20 min), 5.0 μg/ml FHA (purified from B. pertussis BPRA [48] as previously described [49]), 5 μg/ml concanavalin A (Sigma) or medium alone as control. Supernatants were removed from triplicate cultures after 72-h incubation at 37 °C and 5% CO_2_, and IFN-γ and IL-5 concentrations were determined by immunoassays (BD OptEIA set; PharMingen, San Diego, California, United States).

### Statistical analysis.

The results were analyzed using the unpaired Student *t* test and the Kruskal-Wallis test followed by the Dunn post-test (GraphPad Prism program) when appropriate. Differences were considered significant at *p* ≤ 0.05.

## Supporting Information

Figure S1 Lung Colonization and Histological Analysis of Lungs from BPSM- or BPZE1-Infected Infant Mice (A) The 3-wk-old Balb/C mice were infected intranasally with 10^6^ CFU of BPSM (solid lines) or BPZE1 (dotted lines). The results are expressed as mean (± standard error) CFUs from three to four mice per group and are representative of two separate experiments. The dashed line represents the limit of bacterial counts.
(B) Histological analysis of lungs from BPZE1 (upper panel) or BPSM-infected (middle panel) 3-wk-old mice compared to controls given PBS (lower panel). One week after infection, the lungs were aseptically removed and fixed in formaldehyde. Sections were stained with toluidine blue and examined by light microscopy.
(198 KB PPT)

Figure S2 Immune Responses Induced in Adult Mice by BPZE1 or aPV Immunization Anti-FHA (A), anti-PTX (B), anti-B. pertussis IgG heavy and light chain (H+L) titers (C), and anti-FHA IgG1/IgG2a ratios (D) before (open columns) or 1 wk after BPSM challenge (filled columns) in BPZE1 or aPV immunized 8-wk-old mice, compared to controls. Antibodies were measured in individual mice, and the results are expressed as mean values (± standard error) for four to five mice per group.
(46 KB PPT)

Protocol S1 Supplementary Materials and Methods (30 KB DOC)

## Abbreviations

aPV: acellular pertussis vaccine
CFU: colony-forming units
DNT: dermonecrotic toxin
FHA: filamentous hemagglutinin
IgG: immunoglobulin G
ip: intraperitoneal
PTX: pertussis toxin
TCT: tracheal cytotoxin
