# Supplementary material for: Live Attenuated B. pertussis as a Single-Dose Nasal Vaccine against Whooping Cough
Source: PLoS Pathog. 2006 Jul 7;2(7):e65. doi: 10.1371/journal.ppat.0020065 (PMC1487175; doi:10.1371/journal.ppat.0020065)
Supplement: Supplementary file 1 [file ppat.0020065.sd001.doc]

Live Attenuated *B. pertussis* as a Single-Dose Nasal Vaccine against Whooping Cough

Running head: Attenuated Live Pertussis Vaccine

Supplementary material

Nathalie Mielcarek^1^, Anne-Sophie Debrie^1^, Dominique Raze^1^, Julie Bertout^1^, Carine Rouanet^1^, Amena Ben Younes^2^, Colette Creusy^3^, Jacquelyn Engle^4^, William E. Goldman^4^, and Camille Locht^1^*

^1^INSERM U629, ^2^IFR142, Institut Pasteur de Lille, 1, rue du Prof. Calmette, F-59019 Lille Cedex, France; ^3^Service d’Anatomie Pathologique, Hôpital St. Vincent, Bvd. de Belfort, F-59020 Lille Cedex, France, ^4^Washington University, St. Louis, MO, USA

*To whom correspondence should be addressed. E-mail: camille.locht@pasteur-lille.fr; Tel (+33) 3 20 87 11 51; Fax (+33) 3 20 87 11 58

**Supplementary Materials and Methods**

**Construction of *B. pertussis* BPZE1.** To construct *B. pertussis* BPZE1, we first replaced the *B. pertussis* *ampG* gene by *Escherichia coli* *ampG* using allelic exchange. A PCR fragment named *met* and located at position 49,149 to 49,990 of the *B. pertussis* genome (http://www.sanger.ac.uk/Projects/B_pertussis/), upstream of the *B. pertussis* *ampG* gene, was amplified using oligonucleotides A : 5’-TATAAATCGATATTCCTGCTGGTTTCGTTCTC-3’ and B : 5’-tatagctagcaagttgggaaacgacaccac-3’, and *B. pertussis* BPSM [1] genomic DNA as template. This 634-bp fragment was inserted into Topo PCRII (InVitrogen Life Technology, Groningen, The Netherlands) and then excised as a *Cla*I-*Nhe*I fragment and inserted into *Cla*I- and *Nhe*I-digested pBP23 [2], a suicide vector containing the *E. coli* a*mpG* gene with flanking *B. pertussis* DNA of 618 bp (from position 50,474 to 51,092 of the *B. pertussis* genome) and 379 bp (from position 52,581 to 52,960 of the *B. pertussis* genome) at the 5’ and 3’ end of *E. coli* *ampG*, respectively. The resulting plasmid was transferred into *E. coli* SM10 [3], which was then conjugated with BPSM, and two successive homologous recombination events were selected as described [4]. Ten individual colonies were screened by PCR as follows. The colonies were suspended in 100 µl H_2_O, heated for 20 min. at 95°C, and centrifuged for 5 min at 15,000 x *g*. One µl of supernatants was then used as template for PCR using oligonucleotides A and C : 5’-TAAGAAGCAAAATAAGCCAGGCATT-3’ to verify the presence of *E. coli* *ampG* and using oligonucleotides D : 5’-TATACCATGGCGCCGCTGCTGGTGCTGGGC-3’ and E : 5’-tatatctagacgctggccgtaaccttagca-3’ to verify the absence of *B. pertussis* *ampG*. One of the strains containing *E. coli* *ampG* and lacking *B. pertussis* *ampG* was then selected, and the entire *ampG* locus was sequenced. This strain was then used for further engineering.

The *ptx* genes were deleted from the chromosome of this strain as described [5] and then replaced by mutated *ptx* coding inactive PTX. The *Eco*RI fragment containing the mutated *ptx* locus from pPT-RE [6] was inserted into the *Eco*RI site of pJQ200mp18rpsl [7]. The resulting plasmid was integrated into the *B. pertussis* chromosome at the *ptx* locus by homologous recombination after conjugation via *E. coli* SM10. The *ptx* locus in the chromosome of the resulting *B. pertussis* strain was sequenced to confirm the presence of the desired mutations. Toxin production was analyzed by immunoblotting using a mix of monoclonal antibodies IB7 [8] specific for subunit S1, and 11E6 [9] specific for subunits S2 and S3 of PTX.

Finally, the *dnt* gene was deleted from the resulting *B. pertussis* strain as follows. The *dnt* flanking regions were amplified by PCR using BPSM genomic DNA as template and oligonucleotides F : 5’-TATAGAATTCGCTCGGTTCGCTGGTCAAGG-3’ and G : 5’-TATATCTAGAGCAATGCCGATTCATCTTTA-3’ for the *dnt* upstream region, and H 5’-TATATCTAGAGCGGCCTTTATTGCTTTTCC-3’ and I: 5’-TATAAAGCTTCTCATGCACGCCGGCTTCTC-3’ for the *dnt* downstream region, as primers. The resulting 799-bp and 712-bp DNA fragments were digested with *Eco*RI/*Xba*I and *Xba*I/*Hin*dIII, respectively, and linked together using the Fast Link kit (Epicentre Biotechnologies, Madison, WI). The ligated fragment was amplified by PCR using oligonucleotides F and I, and the 1505-bp PCR fragment was then inserted into pCR2.1-Topo (Invitrogen), re-isolated from the resulting plasmid as an *Eco*RI fragment and inserted into the unique *Eco*RI site of pJQmp200rpsL18. The resulting plasmid was introduced into *B. pertussis* by conjugation via *E. coli* SM10. Successful deletion of the *dnt* gene by allelic exchange was verified by Southern blot analysis on *Pvu*II-digested *B. pertussis* genomic DNA using the PCR fragment corresponding to the *dnt* upstream region as a probe. The probe was labeled with digoxigenin (DIG) using the DIG Easy Hyb labeling kit (Roche, Meylan, France). The sizes of the hybridizing bands were determined from the migration distance of the Dig-labeled DNA molecular marker III (Roche). The *dnt* locus of this final strain, named BPZE1 was sequenced.

**REFERENCES**

1 Menozzi FD, Mutombo R, Renauld G, Gantiez C, Hannah JH, *et al*. (1994) Heparin-inhibitable lectin activity of the filamentous hemagglutinin adhesin of *Bordetella pertussis*. Infect Immun 62: 769-778.

2 Lyon RS, Engle JT, Goldman WE. Manuscript in preparation

3 Simon R, Priefer U, Pühler A (1983) A broad host range mobilization system for *in vivo* genetic engineering: transposon mutagenesis in Gram-negative bacteria. Bio/Technology 1: 784–791.

4 Stibitz S (1994) Use of conditionally counterselectable suicide vectors for allelic exchange. Methods Enzymol 235: 458–465.

5 Antoine R, Locht C (1990) Roles of the disulfide bond and the carboxy-terminal region of the S1 subunit in the assembly and biosynthesis of pertussis toxin. Infect Immun 58: 1518-1526.

6 Alonso S, Pethe K, Mielcarek N, Raze D, Locht C (2001) Role of ADP-ribosyltransferase activity of pertussis toxin in toxin-adhesin redundancy with filamentous hemagglutinin during *Bordetella pertussis* infection. Infect Immun 69: 6038-6043.

7 Antoine R, Huvent I, Chemlal K, Deray I, Raze D, *et al*. (2005) The periplasmic binding protein of tripartite tricarboxylate transporter is involved in signal transduction. J Mol Biol 351: 799-809.

8 Sato H, Ito A, Chiba J, Sato Y (1984) Monoclonal antibodies against pertussis toxin: effect on toxin activity and pertussis infections. Infect Immun 46: 422-428.

9 Sato H, Sato Y, Ito A, Ohishi I (1987) Effect of monoclonal antibody to pertussis toxin on toxin activity. Infect Immun 55: 909-915.

**Supplementary Figures**

Figure 1. Lung colonization and histological analysis of lungs from BPSM- or BPZE1-infected infant mice. (a) Three-weeks old Balb/C mice were infected intranasally with 10^6^ CFU of BPSM (black lines) or BPZE1 (dotted lines). The results are expressed as mean (± standard error) CFUs from three to four mice per group and are representative of two separate experiments. The dashed line represents the limit of bacterial counts.. (b) Histological analysis of lungs from BPZE1 (upper panel) or BPSM-infected (middle panel) 3-weeks-old mice compared to controls given PBS (lower panel). One week after infection, the lungs were aseptically removed and fixed in formaldehyde. Sections were stained with toluidin blue and examined by light microscopy.

Figure 2. Immune responses induced in adult mice by BPZE1 or aPV immunization. (a) Anti-FHA, (b) anti-PTX, (c) anti-*B. pertussis* IgG(H+L) titers and (d) anti-FHA IgG1/IgG2a ratios before (white columns) or 1 week after BPSM challenge (black columns) in BPZE1 or aPV immunized 8-weeks old mice, compared to controls. Antibodies were measured in individual mice, and the results are expressed as mean values (± standard error) for 4 to 5 mice per group.
